# Three New Phenolics and Other Constituents from the Seeds of *Lithocarpus pachylepis*

**DOI:** 10.3390/molecules180910397

**Published:** 2013-08-28

**Authors:** Yong Xie, Guoxu Ma, Hua Wei, Jingquan Yuan, Haifeng Wu, Xiaolei Zhou, Junshan Yang, Xudong Xu

**Affiliations:** 1 Key Laboratory of Bioactive Substances and Resources Utilization of Chinese Herbal Medicine, Ministry of Education, Institute of Medicinal Plant Development, Peking Union Medical College and Chinese Academy of Medical Sciences, Beijing 100193, China; E-Mails: yxie@implad.ac.cn (Y.X.); mgxfl8785@163.com (G.M.); hfwu@implad.ac.cn (H.W.); jsyang@implad.ac.cn (J.Y.); 2 Institute of Chinese Materia Medica, China Academy of Chinese Medical Sciences, Beijing 100700, China; E-Mail: weihua20@126.com; 3 National Engineering Laboratory of Southwest Endangered Medicinal Resources Development, National Development and Reform Commission, Guangxi Botanical Garden of Medicinal Plant, Nanning 530023, China; E-Mails: yjqgx@163.com (J.Y.); zhouxiaolei123@163.com (X.Z.)

**Keywords:** Fagaceae, *Lithocarpus pachylepis* A Camus, phenolics, anti-inflammatory activities

## Abstract

Twelve phenolics, including the three new compounds balanophonin C (**1**), balanophonin D (**2**), balanophonin E (**3**), were isolated from the seeds of *Lithocarpus pachylepis*. Their structures were elucidated by various spectroscopic techniques (UV, IR, MS, 1D and 2D NMR). Compounds **1**–**9** were evaluated for their anti-inflammatory activities on lipopolysaccharide (LPS)-induced nitric oxide (NO) production in RAW 264.7 and showed moderate inhibitory activities, with IC_50_ values ranging from 10.9 to 34.7 μM.

## 1. Introduction

**Figure 1:**
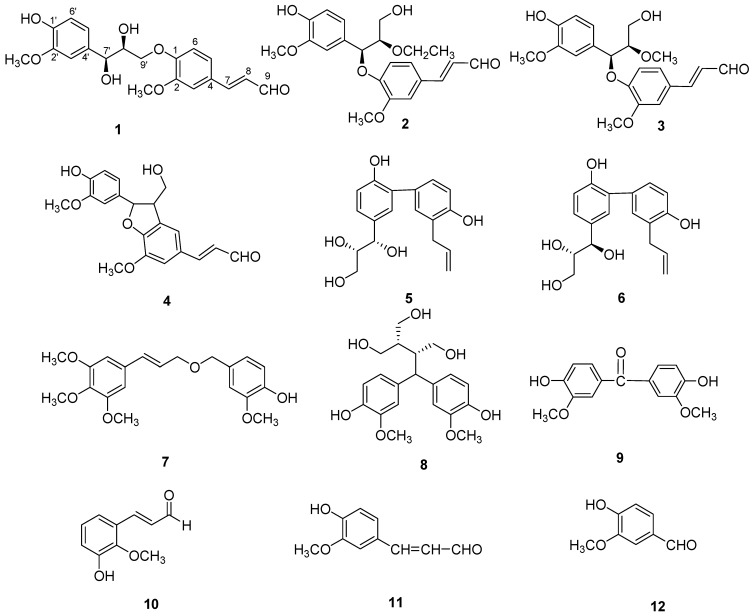
Structures of compounds **1**–**12**.

*Lithocarpus pachylepis* A. Campus is a member of the family Fagaceae. The plant, which grows in the southeast area of China, especially Nanning city of Guangxi Province, is very rare. In Guangxi folk medicine, the seeds of the *Lithocarpus pachylepis*, known as “fengliuguo”, have long been used for the treatment of scapulohumeral periarthritis, impotence, anemia, and hypertension [1]. However, the material basis by which the seeds can treat disease is still unclear. In order to find the bioactive compounds in this plant, our laboratory examined the ethanol extract of its seeds and isolated twelve phenolics, namely balanophonin C (**1**), balanophonin D (**2**), balanophonin E (**3**) balanophonin (**4**) [2], *threo*-honokitriol (**5**) [3], *erythro*-honokitriol (**6**) [3], 2-methoxy-4-[3′-(3′′,4′′,5′′trimethoxyphenyl)allyloxymethyl]phenol (**7**) [4], daphneresinol (**8**) [5], 4,4’-dihydroxy-3,3’-dimethoxybenzophenone (**9**) [6], cassiferaldehyde (**10**) [7], coniferaldehyde (**11**), and vanillin (**12**) (Figure 1). Among them, compounds **1**–**3** are new compounds, the others being isolated from the genus *Lithocarpus* for the first time. In this paper, we report the isolation, structural elucidation, and their inhibitory activities against LPS-induced NO production in macrophages.

## 2. Results and Discussion

Compound **1** was obtained as yellow amorphous powder with 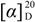 −0.2 (*c* = 0.10, MeOH). It possessed the molecular formula C_20_H_22_O_7_, as revealed by its HR-ESI-MS (*m/z*: 397.1279 [M + Na]^+^, calcd: 397.1263). The IR spectrum showed a hydroxyl absorption at 3,518 cm^−1^ and an *α*,*β*-unsaturated CHO group at 1,670 cm^−1^. A bathochromic shift was observed upon addition of alkali, indicating that the presence of a phenolic benzenoid moiety [5]. The ^1^H-NMR spectrum of **1** (Table 1) showed the presence of two ABX system aromatic rings [*δ*_H_ 7.22 (d, *J* = 1.8), 7.16 (dd, *J* = 8.4, 1.8), 7.01 (d, *J* = 8.4); *δ*_H_ 7.04 (d, *J* = 1.8), 6.85 (dd, *J* = 8.4, 1.8), 6.71 (d, *J* = 8.4)], two methoxyl groups (*δ*_H_: 3.78, 3.83), one *trans*-configuration double bond (*δ*_H_: 7.58, d, *J* = 15.6; 6.65, dd, *J* = 15.6, 7.8) and one aldehyde group (*δ*_H_: 9.58, d, *J* = 7.8). 

**Table 1:** ^1^H- and ^13^C-APT data of compounds **1**–**3 **in CD_3_OD (*δ* in ppm, *J* in Hz).

Position	1		2		3	
	*δ*_C_	*δ*_H_	*δ*_C_	*δ*_H_	*δ*_C_	*δ*_H_
1	152.9		153.0		153.2	
2	152.0		152.1		152.1	
3	113.1	7.22 (d, 1.8)	113.1	7.21 (d, 1.8)	113.0	7.21 (d, 1.8)
4	129.5		129.4		129.6	
5	124.6	7.16 (dd, 8.4, 1.8)	124.5	7.14 (dd, 8.4, 1.8)	124.2	7.16 (dd, 8.4,1.8)
6	117.5	7.01 (d, 8.4)	116.4	6.98 (d, 8.4)	116.5	6.94 (d, 8.4)
7	155.6	7.58 (d, 15.6)	155.6	7.57 (d, 15.6)	155.6	7.54 (d, 15.6)
8	127.9	6.65 (dd, 15.6, 7.8)	127.8	6.67 (dd, 15.6, 7.8)	127.8	6.67 (dd, 15.6,7.8)
9	196.3	9.58 (d, 7.8)	196.2	9.59 (d, 7.8)	196.4	9.60 (d, 7.8)
1′	147.3		147.3		147.3	
2′	148.9		149.1		149.0	
3′	112.3	7.04 (d, 1.8)	112.8	6.96 (d, 1.8)	112.8	6.96 (d, 1.8)
4′	134.2		131.6		131.2	
5′	121.4	6.85 (dd, 8.4, 1.8)	117.4	6.82 (dd, 7.8, 1.8)	117.4	6.82 (dd, 7.8,1.8)
6′	115.8	6.71 (d, 8.4)	115.9	6.74 (d, 7.8)	115.9	6.76 (d, 7.8)
7′	74.4	4.82 (d, 3.6)	76.0	4.50 (d, 3.6)	79.5	4.55 (d, 3.6)
8′	85.6	4.55 (m)	84.9	4.61 (m)	85.2	4.64 (m)
9′	72.7	3.50 (dd, 12.0, 5.4)	62.8	3.48 (m)	62.6	3.47 (m)
		3.85 (dd, 12.0, 3.6)		3.89 (m)		3.86 (m)
2-OCH_3_	56.9	3.83 (s)	56.8	3.83 (s)	56.7	3.81 (s)
2′-OCH_3_	56.6	3.78 (s)	56.6	3.80 (s)	56.6	3.79 (s)
8′-OCH_2_CH_3_			65.2	3.42 (m); 3.85 (m)		
8′-OCH_2_CH_3_			15.8	1.17 (t, 7.2)		
8′-OCH_3_					51.9	3.15 (s)

**Figure 2:**
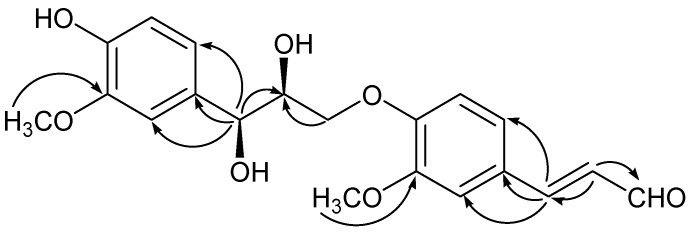
The key HMBC correlations of compound **1**.

The ^13^C-APT spectrum of **1** (Table 1) exhibited 20 carbons, including 14 olefinic carbons, one aldehyde group (*δ*_C_: 196.3), two methoxyl groups (*δ*_C_: 56.6, 56.9), two oxygenated methines (*δ*_C_: 74.4, 85.6) and one oxygenated methylene (*δ*_C_: 72.7). The ^1^H-NMR and ^13^C-APT spectra suggested compound **1 **was a lignan composed of cinnamyl alcohol and cinnamaldehyde [7,8]. In the HMBC spectrum (Figure 2), the correlations from *δ*_H_ 3.50 (H-9', dd, *J* = 12.0, 5.4); 3.85 (H-9', dd, *J* = 12.0, 3.6) to *δ*_C_: 152.1 (C-1) indicated the C-9' was attached to a phenolic O atom; the correlations from *δ*_H_ 7.58 (H-7, d, *J* = 15.6) to *δ*_C_ 113.1 (C-3), 129.5 (C-4), 124.6 (C-5); *δ*_H_ 4.82 (H-7', d, *J* = 3.6) to *δ*_C_ 112.3 (C-3'), 134.2 (C-4'), 121.4 (C-5') indicated that the *trans* double bond was attached to C-4 and the three-carbon chain was linked to C-4'. In addition, the HMBC correlations from *δ*_H_ 3.83 (3H, s, -OCH_3_) to *δ*_C_ 152.0 (C-2); *δ*_H_ 3.78 (3H, s, -OCH_3_) to *δ*_C_ 148.9 (C-2') suggested that the methoxy groups were located at C-2 and C-2'. The relative configuration of **1** was obtained through analysis of coupling constants and the NOESY spectrum. H-7' and H-8' were both determined to be α-oriented on the basis of the NOE enhancement of H-7'/H-8' and the small coupling constant (*J* = 3.6 Hz) between H-7' and H-8'. Therefore, compound **1** was identified as 1',7',8'-trihydroxy-2,2'-dimethoxy-1,9'-oxo-phenylpropylconiferaldehyde and named balanophonin C.

Compound **2** was isolated as yellow amorphous powder. Its molecular formula was deduced as C_22_H_26_O_7_ from HR-ESI-MS (*m/z*: 425.1564 [M + Na]+, calcd: 425.1576). The ^1^H-NMR and ^13^C-APT spectra (Table 1) were similar to those of balanophonin A, except for the extra ethoxyl signals [*δ*_H_: 3.42 (1H, m), 3.85 (1H, m), 1.17 (3H, t, *J* = 7.2); *δ*_C_: 65.2, 15.8] [9]. In the HMBC spectrum, the signals at *δ*_H_: 3.42 (1H, m), 3.85 (1H, m) had direct correlations to *δ*_C_: 62.8 (C-9'), 76.0 (C-7'), 84.9 (C-8') suggested that the ethoxyl group was located at C-8’. Otherwise, the HMBC correlations from *δ*_H_: 4.50 (H-7', d, *J* = 3.6) to *δ*_C_: 112.8 (C-3'), 131.6 (C-4'), 117.4 (C-5'), 153.0 (C-1) indicated the C-7' was attached to the C atom of one aromatic ring and the O atom of another phenolic ether. Combined with the NOESY spectrum, compound **2** was established as 1',9'-dihydroxy-2,2'-dimethoxy-8' -ethoxy-1,7’-oxo-phenylpropylconiferaldehyde and it was named balanophonin D.

Compound **3**, a yellow amorphous powder, was assigned as C_2__1_H_24_O_7_ on the basis of its positive HR-ESI-MS (*m/z* 411.1416 [M + Na]^+^). Comparison of the NMR data (Table 1) between **3** and balanophonin A indicated that compound **3** was another derivative of balanophonin A [9]. The only difference lies in that the hydroxy group at C-8' in balanophonin A was replaced by a methoxy group in **3**. This was fully confirmed by the HMBC correlations from *δ*_H_ 3.15 (8'-OCH_3_, s) to *δ*_C_ 85.2 (C-8'). The similar NOE for **3** and balanophonin A suggested that their relative configuration were identical. Accordingly, the structure of **3** was identified as 1',9'-dihydroxy-2,2'-dimethoxy-8'-methoxy-1,7'-oxo-phenylpropylconiferaldehyde and it was named balanophonin E.

Considering this medicinal herb as a therapeutical agent for the treatment of scapulohumeral periarthritis, the isolated compounds **1**–**9** were studied for their anti-inflammatory activities on lipopolysaccharide (LPS)-induced nitric oxide (NO) production in RAW 264.7. The results indicated that compounds **1**–**9** show moderate inhibitory activities, with IC_50_ values ranging from 10.9 to 34.7 *μ*M (Table 2). From the biological results, it can be inferred that anti-inflammatory activities of the isolated compounds may be partially due to their phenolic structure, while neoligans prevented NO production maybe by suppressing the activation of NF-κB or protein tyrosine phosphorylation reported in previous research [10].

**Table 2:** Inhibitory Activity of Compounds **1**–**9** on LPS-Induced NO Production in RAW 264.7 Macrophages.

Compounds	IC_50_ (μM)
**1**	16.4 ± 1.1
**2**	10.9 ± 0.6
**3**	11.8 ± 0.3
**4**	24.5 ± 2.5
**5**	34.7 ± 0.8
**6**	29.8 ± 1.7
**7**	27.1 ± 3.6
**8**	12.3 ± 1.2
**9**	21.5 ± 1.4
**Aminoguanidine *^a^***	6.8 ± 0.4

*^a^* Positive control substance.

## 3. Experimental

### 3.1. General

Optical rotations were obtained on a Perkin-Elmer 341 digital polarimeter. UV and IR spectra were recorded on a Shimadzu UV2550 and FTIR-8400S spectrometer, respectively. One-dimensional (^1^H, ^13^C-APT) and two-dimensional (^1^H-^1^H COSY, HSQC, HMBC) NMR experiments were performed on a Bruker AV Ш 600 spectrometer operating at 600 MHz (^1^H) and 150 MHz (^13^C). HR-ESIMS spectra were performed on a LTQ-Obitrap XL spectrometer. The detection of all the compounds was achieved in ESI modes. C_18_ reversed-phase silica gel (40–63 μm, Merck, Darmstadt, Germany), Sephadex LH–20 (Pharmacia, Uppsala, Sweden) were used for the column chromatography. Precoated silica gel of GF_254_ plates (Zhi Fu Huang Wu Pilot Plant of Silica Gel Development, Yantai, China) were used for TLC. All solvents used were of analytical grade (Beijing Chemical Works, China). Preparative HPLC was performed on a LUMTECH instrument with UV detector at 254 nm and using an YMC-Pack C_18_ column (250 mm × 20 mm inside diameter (I.D), 5 μm, YMC, Tokyo, Japan).

### 3.2. Plant Material

The seeds of *L. pachylepis* were collected in November 2011 from Nanning, Guangxi Province, China, and identified by Prof. Jing-Quan Yuan, Department of Pharmaceutical Chemistry, Guangxi Botanical Garden of Medicinal Plants, where a voucher specimen (No. 21700) was deposited.

### 3.3. Extraction and Isolation

The air-dried and powdered seeds of *L. pachylepis* (0.7 kg) were refluxed three times with ethanol (3 × 6 L) at 50 °C under reduced pressure. After concentration under reduced pressure, the ethanol extract (16 g) was subjected to column chromatography on silica gel eluting with hexane, chloroform, ethyl acetate, acetone and methanol, respectively (3 × 1,000 mL each). The ethyl acetate fraction (1.8 g) was subjected to reverse phase C-18 chromatography with a gradient of MeOH-H_2_O system (40:60; 50:50; 60:40; 80:20; 100:0) as eluents, yielding five fractions (Fr. 1–5). Fr. 4 (0.31 g) was separated over the Sephadex LH-20 (1 × 45 cm) to remove pigments, then the fraction was subjected to column chromatography on MCI GEL with MeOH-H_2_O system, yielding three fractions (Fr. 4.1–4.3). All the three fractions (Fr. 4.1–4.3) were subjected to HPLC on a Kromasil column. Finally, compounds **2** (2.6 mg) and **4** (4.1 mg) were obtained from fraction 4.1 using a MeOH-H_2_O (38:72) system. Compounds **1** (3.0 mg), **5** (1.8 mg) and **6** (2.9 mg) were obtained from fraction 4.2 using a MeOH-H_2_O (46:54) system. Compounds **3** (2.7 mg) and **8** (5.3 mg) was obtained from fraction 4.3 using a MeOH-H_2_O (50:50) system. Fr. 5 (0.18 g) was separated over Sephadex LH-20 and MCI GEL respectively, yielding two fractions (Fr. 5.1–5.2). These two fractions were subjected to HPLC on a Kromasil column. Finally, compound **7** (4.5 mg) and **9** (6.5 mg) were obtained from fraction 5.1 using a MeOH-H_2_O (52:48) system. Compound **10** (4.7 mg), **11** (5.9 mg) and **12** (3.7 mg) were obtained from fraction 5.2 using a MeOH-H_2_O (58:42) system.

### 3.4. Spectral Data

Compound **1**: Yellow amorphous powder. 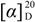 −0.2 (*c* = 0.10, MeOH). UV *λ*_max_ (MeOH) nm (log *ε*): 285 (3.15), 256 (3.78), 212 (4.16). IR (KBr) cm^−1^ 3518, 1670. ^1^H and ^13^C-APT (CD_3_OD): See Table 1. HR-ESI-MS *m/z*: 397.1279 [M + Na]^+^ (Calcd for 397.1263).

Compound **2**: Yellow amorphous powder. 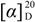 −0.38 (*c* = 0.09, MeOH). UV *λ*_max_ (MeOH) nm (log *ε*): 285 (3.42), 256 (4.01), 212 (4.62). IR (KBr) cm^−1^ 3524, 1674. ^1^H and ^13^C-APT (CD_3_OD): See Table 1. HR-ESI-MS *m/z*: 425.1564 [M + Na]^+^ (Calcd for 425.1576).

Compound **3**: Yellow amorphous powder. 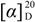 −0.36 (*c* = 0.08, MeOH). UV *λ*_max_ (MeOH) nm (log *ε*): 285 (3.23), 256 (3.61), 212 (4.04). IR (KBr) cm^−1^ 3520, 1672. ^1^H and ^13^C-APT (CD_3_OD): See Table 1. HR-ESI-MS *m/z*: 411.1416 [M + Na]^+^ (Calcd for 411.1420).

The structures of compounds **4**–**12** were identified by comparison of their spectral data with those reported in the literature.

## 4. Conclusions

Three new phenolics named balanophonin C (**1**), balanophonin D (**2**), balanophonin E (**3**), together with nine known phenolics balanophonin (**4**) [2], *threo*-honokitriol (**5**) [3], *erythro*-honokitriol (**6**) [3], 2-methoxy-4-[3'-(3'',4'',5''trimethoxyphenyl)allyloxymethyl]phenol (**7**) [4], daphneresinol (**8**) [5], 4,4'-dihydroxy-3,3'-dimethoxybenzophenone (**9**) [6], cassiferaldehyde (**10**) [7], coniferaldehyde (**11**), and vanillin (**12**) were isolated from the seeds of *L. pachylepis*. The isolation of the new compounds is an addition to the molecular diversity of *L. pachylepis*. All the isolated compounds showed moderate anti- inflammatory activities.
